# Periconceptional Maternal Diet Characterized by High Glycemic Loading Is Associated with Offspring Behavior in NEST

**DOI:** 10.3390/nu13093180

**Published:** 2021-09-13

**Authors:** Candice L. Alick, Rachel L. Maguire, Susan K. Murphy, Bernard F. Fuemmeler, Cathrine Hoyo, John S. House

**Affiliations:** 1 Center for Health Promotion and Disease Prevention, University of North Carolina at Chapel Hill, Chapel Hill, NC 27514, USA; candice.alick@gmail.com; 2 Center for Human Health and the Environment, North Carolina State University, Raleigh, NC 27695, USA; rlmaguir@ncsu.edu (R.L.M.); choyo@ncsu.edu (C.H.); 3 Department of Biological Sciences, North Carolina State University, Raleigh, NC 27695, USA; 4 Department of Obstetrics and Gynecology, Duke University Medical Center, Durham, NC 27701, USA; susan.murphy@duke.edu; 5 Department of Health Behavior and Policy, Virginia Commonwealth University, Richmond, VA 23219, USA; Bernard.fuemmeler@vcuhealth.org; 6 Biostatistics and Computational Biology Branch, National Institute of Environmental Health Sciences, National Institutes of Health, Department of Health and Human Services, Research Triangle Park, Durham, NC 27709, USA

**Keywords:** maternal diet, neurodevelopment, cord blood methylation, child behavior disorders, ADHD attention-deficit disorder, autism spectrum disorder, epigenetics, imprinted genes, glycemic index, glycemic loading

## Abstract

Maternal periconceptional diets have known associations with proper offspring neurodevelopment. Mechanisms for such associations include improper energy/nutrient balances between mother and fetus, as well as altered offspring epigenetics during development due to maternal nutrient and inflammatory status. Using a comprehensive food frequency questionnaire and assessing offspring temperament with the Infant-Toddler Social and Emotional Assessment (*n* = 325, mean age = 13.9 months), we sought to test whether a maternal periconceptional diet characterized by high glycemic loading (MGL) would affect offspring temperament using adjusted ordinal regression. After limiting false discovery to 10%, offspring born to mothers in tertile 3 of glycemic loading (referent = tertile 1) were more likely to be in the next tertile of anxiety [OR (95% CI) = 4.51 (1.88–11.07)] and inhibition-related behaviors [OR (95% CI) = 3.42 (1.49–7.96)]. Male offspring were more likely to exhibit impulsive [OR (95% CI) = 5.55 (1.76–18.33)], anxiety [OR (95% CI) = 4.41 (1.33–15.30)], sleep dysregulation [OR (95% CI) = 4.14 (1.34–13.16)], empathy [6.68 (1.95–24.40)], and maladaptive behaviors [OR (95% CI) = 9.86 (2.81–37.18)], while females were more likely to exhibit increased anxiety-related behaviors [OR (95% CI) = 15.02 (3.14–84.27)]. These associations persisted when concurrently modeled with the maternal–Mediterranean dietary pattern. In a subset (*n* = 142), we also found MGL associated with increased mean methylation of the imprint control region of *SGCE/PEG10*. In conclusion, these findings highlight the importance of maternal dietary patterns on offspring neurodevelopment, offering avenues for prevention options for mothers.

## 1. Introduction

Maternal diet and nutrition are integral to proper neurodevelopment in offspring and have been shown to affect the brain health of both term and preterm offspring [1]. An unhealthy maternal diet has been linked to offspring neurodevelopmental disorders [2].

Children born of mothers who were more adherent to the Mediterranean diet, long lauded for its benefits on cardiovascular disease and aging-related neurocognitive decline, were less likely to exhibit adverse behavioral outcomes from depression, anxiety, atypical behaviors, maladaptive behaviors and were more likely to score higher in the social-relatedness index [3]. Other favorable diets, characterized by consumption of carbohydrates from whole grains, fruits, vegetables, and milk, have been associated with low glycemic loading when compared to diets high in carbohydrates from bread and desserts [4]. An in-depth review and meta-analysis provides evidence for associations between better maternal diet quality and offspring neuro- and cognitive development [5].

Glycemic loading, which accounts for both glycemic index and total carbohydrate intake, provides a more precise indicator of dietary impact on blood sugar than the glycemic index. Glycemic index represents the rate at which a food item causes an increase in blood glucose levels. Glycemic load, however, reflects both the glycemic index of a food item as well as the amount of carbohydrate loading per serving.

Higher maternal glycemic loading (MGL) in early pregnancy was associated with greater adiposity in childhood [6]. In mid-pregnancy, a sample of Danish pregnant women reporting higher maternal dietary glycemic load also reported a higher birth weight and an increased risk of delivering a large-for-gestational-age infant [7]. Likewise, in the Generation R study with 3104 children, a maternal diet high in meat, margarine, and potatoes was associated with increased externalizing behaviors in offspring [8].

In addition to a host of associated human disease and health outcomes (Supplemental Table S1), alterations in the methylation of imprint control regions of imprinted genes have been identified as a possible causal link between early maternal dietary patterns and child temperament and behavior [3,9], as well as lifelong changes in energy metabolism [10]. Increased at-birth-methylation of the imprint control region governing the imprinted insulin-like growth factor 2 (*IGF2*) gene, critical in fetal and placental growth regulation and in proper cerebellum/hippocampal development, was associated with early onset recurring conduct problems in the ALSPAC cohort [11]. The authors also found evidence for mediation of attention deficit hyperactivity disorder behaviors in children with conduct problems via methylation of the *IGF2* control region.

In the current study, we hypothesized that children born of mothers with a periconceptional high glycemic loading dietary pattern would have adverse temperament outcomes. Further, we tested whether this dietary pattern would affect imprint control region methylation in the NEST cohort. Lastly, our previous work reported favorable associations with maternal adherence to a Mediterranean diet (MDA) and offspring behavior—namely, that MDA was associated with favorable child temperament in the depression, anxiety, atypical, and maladaptive indices of the Infant-Toddler Social and Emotional Assessment (ITSEA). We tested whether offspring temperament associations reported herein regarding MGL were independent of those associated previously with MDA.

## 2. Materials and Methods

### 2.1. Cohort and Selection Criteria

Study participants consist of mother/infant dyads enrolled in the Newborn Epigenetics Study (NEST), based in Durham, North Carolina. As described in detail in [3], these analyses assess the same *n* = 325 dyads that completed both a food frequency questionnaire and an Infant-Toddler Social and Emotional Assessment (ITSEA) during the child’s second year of life. As described, mothers’ responses to the food frequency questionnaire (FFQ) concerned dietary food recall during the time of conception, and responses were converted to glycemic index and glycemic loading values by Nutrition Quest (www.nutritionquest.com, accessed on 26 January 2013).

### 2.2. Child Behavioral Assessment

The details of how we assessed behavioral outcomes have been listed in full in [3]. In brief, the ITSEA [12] was administered by caregivers or staff for children between 12 and 24 months of age (mean = 13.9) during a NEST follow-up visit. The ITSEA has been validated and heavily used to assess child temperament in children as young as 11 months of age with excellent inter-rater and test–retest reliability [12,13]. The scoring of the ITSEA scales consists of 0 = Not True/Rarely, 1 = Somewhat, and 2 = Very True/Often, and the instrument uses over 160 questions to assess childhood temperament in broad categories for externalizing, internalizing, dysregulation, competency-related, maladaptive, atypical and social-relatedness behaviors. Competency-related domains and the social-relatedness category are considered favorable outcomes with an increased score, while all others are considered adverse with an increased score. The problem and competency portions of the ITSEA are also grouped into a composite autism spectrum disorder (ASD) category [14]. As before, ITSEA behavioral scores were broken into tertiles and assessed using ordinal logistic regression for associations with maternal diet, and the peer-aggression subscale of Externalization behaviors was not assessed due to extremely limited nonzero scoring (>2/3 data = zero; data not shown) [3].

### 2.3. Glycemic Loading

Pregnant women’s glycemic load was calculated from a food frequency questionnaire (FFQ) completed after enrollment concerning eating habits around their last menstrual period (University of Texas, MD Anderson Cancer Center Nutrition and Lifestyle Core Questionnaire 2008 v.2; modified block questionnaire with additional foods added concerning southeastern eating habits). The questionnaire includes hundreds of data points concerning intake and dosing of supplements and vitamins, followed by extensive questions of fast-food intake and food types. Serving size (denoted with example pictures) and frequency were collected. For example, regarding consumption of chips, questions include frequency per day/week/month/year, and what size bag (snack bag size/grab bag size/½ family size bag). When asking about hamburgers, color photos depicting 5 grades of cooked (rare to well done) are provided. When asking about cereals, color pictures of bowls with different serving sizes are depicted. Data from these surveys were converted to intakes of foods, food groups (i.e., carbohydrates), energy, and nutrients by Nutrition Quest [3].

Glycemic loading for a given food is calculated by the following: Glycemic Load_food_ = (Glycemic Index_food_ × Available Carbohydrate_food_ per serving)/100. This value is multiplied by the frequency of consumption, and the total glycemic load is the sum of the values from all food [15,16,17]. The total glycemic load score was divided into tertiles (referent = first tertile) and used to assess associations of periconceptional maternal glycemic load with child behaviors.

Glycemic loading provides additional information when compared to the glycemic index in that glycemic load also considers the available amount of carbohydrates in a serving of the food consumed—the outcome being a more informative assessment of how food impacts blood glucose level. For example, a watermelon has a glycemic index of 72 (very high) but a glycemic load of 4 in 2 cups of fruit due to being composed primarily of water (90%). In contrast, a medium-baked russet potato has a slightly lower glycemic index of 63 but a much higher glycemic load of over 20.

### 2.4. DNA Methylation Assessment

Complete descriptions are the same as in [3]. In brief, 40 ng of bisulfite-treated DNA was used to assess 48 CpG sites across 8 differentially methylated regions (DMRs) by pyrosequencing for the following imprinted control regions: *IGF2*, *H19*, *MEG3*, *MEG3*-*IG* (regulates *MEG3* and *DKL1*), *NNAT*, *MEST*, *SGCE/PEG10*, and *PLAGL1*. Complete descriptions of these regions, as well as assay conditions and quality control criteria, are found elsewhere [18,19,20,21]. For the assessment of MGL on methylation, the number of subjects with maternal dietary information, all covariates, and cord blood methylation varied.

There are relatively few imprint regulatory regions that have been empirically determined. These regions were chosen because they had previously been well characterized for their methylation status and because they contribute to the normal silencing of one of the two parental alleles for genes within the imprinted domain they regulate. They control the expression of multiple genes associated with a variety of human diseases and health conditions, including those involved in neurodevelopmental outcomes (Supplemental Table S1). Thus, ICRs in general, make good “candidate” areas to study the link between maternal exposures and offspring outcomes.

The first *n* = 620 mothers recruited who consented to cord blood retrieval were the group assessed for methylation values in these regions. The overlap of those with mothers who had answered a food frequency questionnaire and for whom ITSEA was administered to offspring at the one-year follow-up numbered 142 and were the dyads used for examining associations of maternal diet with offspring methylation alterations in this analysis.

### 2.5. Statistical Analysis

We examined associations of MGL on tertiles of child behavioral outcomes with ordinal logistic regression (using *polr*() from MASS package in R) and adjusted for age at delivery, education level (any college vs. none), prepregnancy obesity (BMI > 30 vs. BMI < 30), race (white, black, Hispanic, other), smoking during pregnancy, any of gestational, type I or type II diabetes, daily intake of folate (diet plus supplements: <400 μg, 400–800 μg, >800 μg), daily fiber intake, daily energy intake, and any breastfeeding of at least 3 months. We also adjusted for child sex, child birth weight, full-term status (≥37 weeks gestation or not), parity (nulliparous vs. not), and age of ITSEA assessment. These analyses were repeated stratified by child sex.

To test whether previous findings on Mediterranean diet adherence (MDA) and the findings presented herein on MGL exhibited independent associations on offspring behavior, both dietary patterns were concurrently modeled as predictors for tertiles of behavioral outcomes using ordinal logistic regression while adjusting for the same covariates. For assessment of maternal diet on offspring methylation, child age at ITSEA and breastfeeding status were removed as covariates.

All analyses were carried out using R version 4.1.0 (R Development Core Team. R Foundation for Statistical Computing, Vienna, Austria). Assessment of maternal glycemic load and mean percent methylation of CpGs was conducted with linear regression using lm() from the base R stats package. Where indicated, correction for multiple comparisons was performed by *p.adjust()* in base R using Benjamini–Hochberg methodology [22] while holding true discovery to 90% (FDR ≤ 10%) for each tertile assessed across 20 behavioral outcomes.

## 3. Results

Study demographics, covariate, and temperament distributions are unchanged from those reported in (House et al., 2018). In brief, of *N* = 325 mother/offspring dyads, 53.5% of offspring were male. Roughly 42% of mothers identified as white, 29% as black, and 22% as Hispanic. Full-term births (92%) were predominant, and first-child births represented 44% of offspring. Smoking mothers were few (11%). Mothers without at least some college education represented 37.5%. A minority of mothers were obese (24.5% with BMI ≥ 30). The average age of temperament assessment via the ITSEA was 13.9 months. There were little differences in average height and weight at birth and average monthly gain in weight and height between birth and assessment of temperament with the ITSEA instrument (Table 1).

### 3.1. Associations of Maternal Glycemic Loading Dietary Pattern with Offspring Behavior

**Figure 1:**
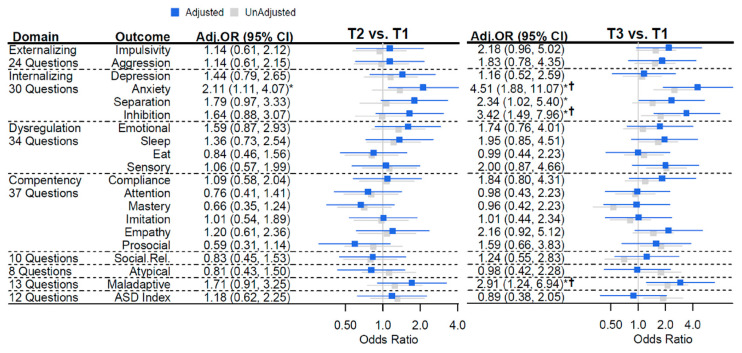
MGL and child temperament. For a given tertile of MGL compared to tertile 1 (referent), the odds ratio (95% confidence interval) represents the risk of being in a higher tertile of behavioral outcome. Unadjusted (gray) and adjusted (blue) odds ratios (95% confidence intervals) are plotted. Estimates were adjusted for breastfeeding at least 3 months, age of child at behavioral assessment, maternal fiber intake, total calories, folate, education, diabetes, obesity, smoking, and age, as well as child parity, premature birth, weight, race, and child sex. * *p* < 0.05; ^†^ Benjamini–Hochberg FDR < 0.10.

We first examined associations between MGL at or near conception with childhood social–emotional behaviors across all 325 dyads while adjusting for child sex. Using the Infant-Toddler Social and Emotional Assessment (ITSEA) among offspring between 12 and 24 months of age and using the lowest MGL tertile as the referent, offspring born to mothers in the highest tertile, were more than 4 times more likely to be in a higher tertile of anxiety [OR (95% CI) = 4.51 (1.88, 11.07)] (Figure 1). Children born to mothers in the highest tertile of MGL were also more likely to exhibit increased separation-related behaviors [OR (95% CI) = 2.34 (1.02, 5.40)], inhibition [OR (95% CI) = 3.42 (1.49, 7.96)], and maladaptive behaviors [2.91 (1.24, 6.94)]. Anxiety, inhibition, and maladaptive related associations were still significant when controlling false discovery ≤ 10%. These same trends were evident in a GL dose–response manner even when comparing the middle tertile of MGL to the referent (lowest), but only anxiety-related behaviors were significant [OR (95% CI) = 2.11 (1.11, 4.07)] (Figure 1).

As the type of daycare can affect child neurodevelopment and temperament, we conducted a sensitivity analysis for the smaller subset of mothers who responded to any type of daycare questions and separated respondents who reported center-based daycare versus other types of daycare (yes = 55, other = 144). Including center-based daycare in the sex-adjusted models (Figure 1) resulted in attenuation of the estimate of anxiety when comparing offspring of mothers in the middle tertile of MGL vs. referent (T2 vs. T1) [OR (95% CI) = 1.74 (0.77, 3.96)], and for comparing offspring of mothers in the highest tertile of MGL vs. referent (T3 vs. T1) [OR (95% CI) = 2.63 (0.86, 8.17)]. Estimates for the likelihood of children born of mothers in T3 versus T1 of MGL to exhibit an increased risk of separation behaviors were no longer significant but materially unchanged [OR (95% CI) = 2.83 (0.94, 8.67)], while for T2 versus T1 mothers, they became significant and were materially unchanged [OR (95% CI) = 2.32 (1.05, 5.20)]. The estimate of inhibition-related behaviors in offspring of T3 vs. T1 of MGL was also materially unchanged [OR (95% CI) = 3.94 (1.31, 12.38)]. Interestingly, including this daycare-related variable in the model increased the odds of a child born to mothers in the T3 vs. T1 of MGL exhibiting compliance-related behaviors [OR (95% CI) = 3.74 (1.17, 12.51)], which is favorable. Lastly, the estimate for the association of maladaptive behaviors in offspring of mothers in T3 vs. T1 of MGL was also attenuated [OR (95% CI) = 1.91 (0.6, 6.13)] and no longer significant when adjusting for center-based daycare.

### 3.2. Associations of MGL Dietary Pattern with Offspring Behavior by Sex

**Figure 2:**
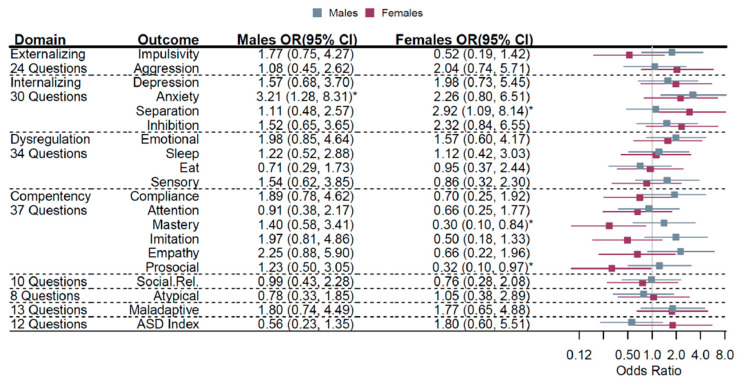
Sex-specific associations of MGL (T2 vs. T1) and offspring temperament. For a given tertile of MGL, compared to tertile 1 (referent), the odds ratio (95% confidence interval) represents the risk of being in a higher tertile of behavioral outcome. Males (gray) and females (magenta) odds ratios (95% confidence intervals) are plotted. Estimates were adjusted as before stratified by sex. * *p* < 0.05.

We next examined associations of MGL on offspring behavioral outcomes by sex of the child. Anxiety [OR (95% CI) = 3.21 (1.28, 8.31)] was more likely among male offspring of mothers in the middle MGL tertile, compared to the lowest tertile. In females, separation [OR (95% CI) = 2.92 (1.09, 8.14)] was more likely, while mastery [OR (95% CI) = 0.30 (0.10, 0.84)] and prosocial [OR (95% CI) = 0.32 (0.10, 0.97)] behaviors (both favorably scaled) were less likely (Figure 2). Although dose–response patterns of association were apparent, none of these associations survived correction for false discovery.

**Figure 3:**
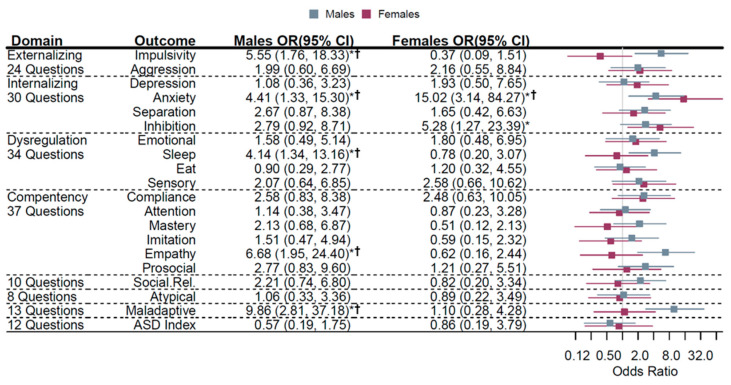
Sex-specific associations of MGL (T3 vs. T1) and offspring temperament. Sex-specific associations with MGL (tertile 3 vs. tertile 1) and offspring behavioral outcomes. For a given tertile of MGL compared to tertile 1 (referent), the odds ratio (95% confidence interval) represents the risk of being in a higher tertile of behavioral outcome. Males (gray) and females (magenta) odds ratios (95% confidence intervals) are plotted. Estimates were adjusted as before stratified by sex. * *p* < 0.05; ^†^ Benjamini–Hochberg FDR < 0.10.

When examining associations of the highest tertile of MGL to lowest with sex-stratified child behavioral outcomes, males had increased odds of being in the next highest tertile of impulsive behaviors [OR (95% CI) = 5.55 (1.76, 18.33)], anxiety [OR (95% CI) = 4.41 (1.33, 15.30)], sleep dysregulation [OR (95% CI) = 4.14 (1.34, 13.16)], empathy [OR (95% CI) = 6.68 (1.95, 24.40)], and maladaptive behaviors [OR (95% CI) = 9.86 (2.81, 37.18)] (Figure 3). All of these were still significant while limiting FDR to 10% or less. Among female offspring, there was an increased odds of being in the next highest tertile of anxiety-related behaviors [OR (95% CI) = 15.02 (3.14, 84.27)] and inhibition-related behaviors [OR (95% CI) = 5.28 (1.27, 23.39)] with the former surviving FDR correction (Figure 3).

### 3.3. Imprinted Control Regions (ICRs) for Nine Imprinted Genes and Multiple CpGs

**Figure 4:**
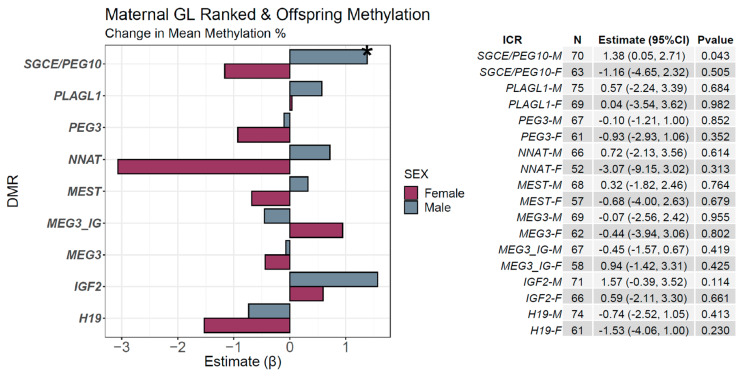
Sex-specific associations of MGL and offspring imprint control region methylation. Sex-specific associations of ICRs and MGL diet. Tertiles of MGL were assessed for associations with mean methylation values at known imprint control regions. For example, each tertile of MGL was associated with a 1.38% increase in mean methylation at the *SGCE/PEG10* ICR. Estimates were adjusted for maternal fiber intake, total energy intake, self-reported race, smoking status, diabetes status, folate intake, age, obesity status, education, as well as paternal age and child birth weight, preterm status, and parity. * *p* < 0.05.

To test mediation effects of methylation of control regions of imprinted genes on the association between MGL and offspring behavior we examined the relationship of MGL on CpG methylation of differentially methylated regions (DMR) regulating nine imprinted genes. The mean methylation percentage of CpGs of the DMRs of the imprinted genes, *SGCE/PEG10*, *PLAGL1*, *PEG3*, *NNAT*, *MEST*, *MEG3 IG*, *MEG3*, *IGF2*, and *H19*, was assessed with a covariate-adjusted multiple linear regression. These data were stratified by sex. In males, a maternal diet with the highest tertile of glycemic loading was associated with an increase in mean methylation of the ICR of *SGCE/PEG10* [β (95% CI) = 1.38 (0.05, 2.71); *p*-value = 0.043] (Figure 4).

### 3.4. Associations of MGL on Offspring Behaviors While Adjusting for Maternal Mediterranean Dietary Pattern

**Figure 5:**
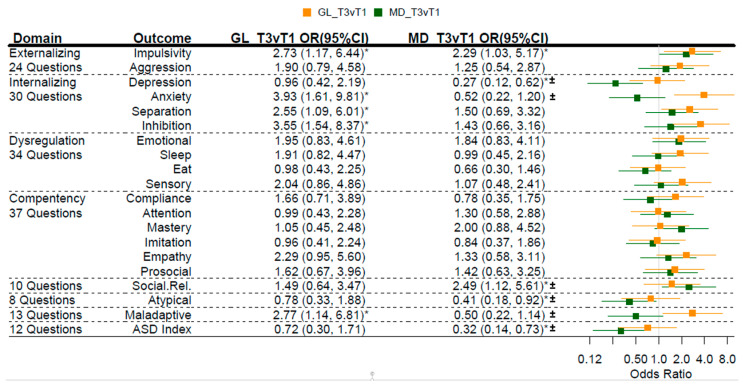
Concurrent modeling of MGL and Mediterranean dietary adherence on offspring temperament. For the 3rd tertile of MGL compared to the 1st (referent), the odds ratio (95% confidence interval) represents the risk of being in a higher tertile of behavioral outcome. Odds ratios (95%CI) are plotted for glycemic loading (GL; orange) and Mediterranean diet (MD; green). Estimates were adjusted for breastfeeding at least 3 months, age of child at behavioral assessment, maternal fiber intake, total calories, folate, education, diabetes, obesity, smoking, race, and age, as well as child parity, premature birth, weight, and child sex. * *p* < 0.05; **^±^** Previously reported significant when modeled without MGL as a concurrent predictor.

Among our 325 mothers, maternal adherence to the Mediterranean dietary pattern and MGL were inversely correlated (rho = −0.37), consistent with one dietary pattern partially substituted for another. To assess whether these reported adverse effects on child behavior were due to MGL or perhaps explained by a commensurate lack of Mediterranean dietary adherence (MDA), we concurrently modeled both maternal dietary patterns adjusting for the same covariates (Figure 5).

In nearly all cases, the observed behavioral associations for MGL persisted. Just as strikingly, significant associations reported on the Mediterranean dietary pattern also persisted when concurrently modeled with MGL (Figure 5), suggesting that associations of offspring behaviors with these two inversely correlated maternal dietary patterns are largely independent of each other. When jointly modeling MGL and maternal adherence to a Mediterranean diet, and using the lowest MGL tertile as the referent, offspring born to mothers in the highest MGL tertile were more likely to score in the highest tertile of impulsivity [OR (95% CI) = 2.73 (1.17, 6.44)], anxiety [OR (95% CI) = 3.93 (1.61, 9.81)], separation [OR (95% CI) = 2.55 (1.09, 6.01)], inhibition [OR (95% CI) = 3.55 (1.54, 8.37)], and maladaptive behaviors [OR (95% CI) = 2.77 (1.14, 6.81)].

Sex differences were also pronounced between MGL and childhood behaviors. In male offspring (Figure S1), when comparing the lowest MGL tertile and the highest tertile, there was an increased likelihood in impulsivity [OR (95% CI) = 6.77 (2.08, 23.25)], and anxiety [OR (95% CI) = 3.60 (1.05, 12.87)]. Among dysregulation behaviors, a similar association was observed between sleep [OR (95% CI) = 4.51 (1.43, 14.77)] and MGL. Offspring born to mothers in the highest MGL tertile were more likely to score in the highest tertile of maladaptive behaviors [OR (95% CI) = 10.47 (2.87, 41.36)] but also empathy [OR (95% CI) = 5.76 (1.66, 21.36)] (Figure S1). In female offspring, those from mothers in the highest MGL were more likely to score in the highest tertiles of anxiety [OR (95% CI) = 14.14 (2.82, 82.00)] and inhibition [OR (95% CI) = 5.16 (1.19, 23.73)] when compared to the lowest MGL tertile. Again, previously reported findings of associations with maternal Mediterranean diet persisted when concurrently modeled with MGL.

## 4. Discussion

We tested the hypothesis that a periconceptional diet marked by high glycemic loading would affect aspects of offspring neurodevelopment and temperament, and further examined whether associations between MGL and offspring temperament may be mediated by dysregulation of genomic imprinting in offspring, detectable at birth in cord-blood. In this analysis, using data from the NEST cohort, we observed marked differences in child temperament scores in relation to maternal diet around the time of conception. When examining the highest tertile of MGL to the lowest, and after adjusting for an FDR of 10% or less, these associations of offspring behavior patterns with MGL were largely adverse with the exception of male children more likely to express greater empathy when the maternal diet was characterized by the highest levels of glycemic loading. Males also had a greater risk of impulsivity, anxiety, maladaptive behaviors, and sleeping issues. Female children expressed an increased risk of anxiety. When examined jointly in the context of a “healthy” maternal Mediterranean diet, previously reported favorable associations with offspring temperament persisted, as did adverse associations with MGL and offspring temperament, indicating these dietary associations with offspring behavioral patterns were largely independent of each other.

When we tested whether MGL was associated with changes in methylation of CpG’s of nine imprint control regions, the results were largely null except for hypermethylation of the CpGs in the *SGCE/PEG10* ICR in males, and these associations did not survive FDR correction. This gene cluster has primarily been associated with proper placental development and cancer [23,24,25,26], but previous work has also implicated hypermethylation of PEG10 with increased offspring weight for length ratios [27]. Further, the *SGCE/PEG10* ICR has been implicated in Silver–Russell syndrome, a rare disorder characterized by improper development of facial features, growth retardation, and developmental delays in speech and motor function [28].

The Generation R cohort, a study of maternal diet and offspring temperament, reported an increase in externalizing behaviors with a maternal diet high in meat, margarine, and potatoes [8]. Multiple animal studies support that MGL can affect offspring neurodevelopment and temperament. Dam rats fed a high sucrose diet during pregnancy had offspring with a decreased brain-to-body-weight ratios, decreased performance on the Morris water maze, and exhibited gene-expression perturbations in the hippocampus, compared to offspring of dams fed standard chow [29]. In an animal model used to study ASD, mice dams fed a ketogenic diet (extremely low MGL) had offspring with ameliorated ASD behaviors [30]. We reported a greater than fourfold increase in the odds of males born to mothers with the highest tertile of MGL being in the next tertile of anxiety-related temperament. An animal study with rats reported similar results from dams fed with excessive caloric intake. Male offspring of such dams also exhibited increased anxiety [31].

Glycemic load describes both the impact a food item containing carbohydrates has on postprandial glucose or blood glucose and insulin levels after consumption and the amount of that food actually consumed (i.e., serving of carbohydrates consumed). This quantification of the impact of carbohydrate-rich foods on blood glucose differs from the glycemic index in that the glycemic index measure ranks foods from 1 to 100. The ranking of foods is calculated with the following formula: incremental area under the curve for blood glucose after consumption of carbohydrate-containing food divided by the incremental area under the curve of a reference food containing the same amount of carbohydrate [17]. Foods with low glycemic indices have lower ranks, and foods with high glycemic indices have higher rankings. Glycemic loads provide additional information when compared to the glycemic index in that glycemic load also considers the available amount of carbohydrates in a serving of the food consumed—the outcome being a more informative assessment of how a food impacts blood glucose levels and subsequent insulin signaling.

Girchenko et al. reported mediation effects from maternal antenatal inflammatory markers on associations of environmental diversities with child developmental delay [32]. A possible mechanism for the associations we found between MGL and offspring temperament may be related to inflammation from a persistent high glycemic load diet. It may be that the long-term effects of a diet characterized by a high-glycemic load and the corresponding inflammation from persistent excessive insulin signaling provide a partial mechanism in explaining these associations. Similarly, recent work by Polanska et al. examined diets in *n* = 11,870 mother/child dyads across four European cohorts and reported associations of adverse offspring emotional and behavioral symptoms with maternal low-quality and proinflammatory diets [33]. Aside from DNA methylation, other types of epigenetic alterations such as histone modifications and placental micro-RNA expression have been both inheritably altered in response to aging, diet, and environmental exposures [34].

Our findings should be interpreted in the context of our study limitations. For example, while our study is adequately powered to evaluate associations between MGL and neurodevelopmental outcomes, we are underpowered to evaluate sex-specific effects. Despite this limitation, multiple sex-specific findings were significant even after limiting FDR to 10%. In addition, we were unable to ascertain if offspring diet affected or moderated the reported associations between mother’s diet and offspring behavior, although due to the mean age at assessment (~14 months), food intakes are less likely to have diverged. Another limitation is that while the use of FFQ to estimate MGL is established, early pregnancy is a time of dynamic dietary changes over very short time periods, and we cannot exclude the possibility that under-reporting of MGL may have influenced our findings. It is critical to point out that mothers were asked to recall their diet at and around the time of conception, to capture extant diet prior to or as early as possible in pregnancy, and prior to dietary changes due to pregnancy. It has been proposed that future studies augment dietary assessments with tools such as Veggie meters^®^ that estimate fruit and vegetable, to reduce misclassification.

Our analysis supports the need for further investigation of the association between maternal (and offspring) diets characterized by high glycemic loading and neurocognitive behaviors, specifically examining sex differences. Sex-specific neurodevelopment outcomes related to other prenatal exposures, such as maternal anxiety, depression, or stress during pregnancy have been noted in other research [33,35], and a number of preclinical studies of prenatal exposure have resulted in sexual dimorphic developmental outcomes [36,37]. These differences could reflect sex-specific placental adaptation to variation in prenatal exposures, such as nutritional quality, and/or differences in neurodevelopmental trajectories between males and females. They may also reflect issues related to measurement and reporter biases; thus, studies examining sex differences would be prudent to ascertain assessments by other reporters besides the mother. With these limitations in mind, our results suggest that during pregnancy, maternal diets characterized by high glycemic loading may promote poor neurocognitive functioning among boys. Although our findings were not significant for the association of increased IGF2 methylation from a high glycemic loading maternal diet, the direction of effect (in male offspring) was the same as in previous findings, highlighting an increase in IGF2 methylation due to an unhealthy maternal diet during gestation (not postnatal) [11]. Furthermore, it appears favorable offspring behaviors associated with maternal adherence to a Mediterranean dietary pattern in and around conception are independent of the adverse offspring outcomes reported in this work from a maternal diet high in glycemic loading. In total, these data highlight both the importance of maternal diet on offspring temperament and the need for future larger studies to elucidate further epigenetic mechanisms.

## Figures and Tables

**Table 1:** Offspring weight and height statistics.

Tertile of MGL (*N*)	Birth Weight-kg	*N*	Birth Height-cm	*N*	Weight Gain Per Month-kg	*N*	Height Gain Per Month-cm	*N*
1	3.29 (0.59)	107	50.09 (3.29)	91	0.53 (0.10)	91	2.02 (0.33)	69
2	3.40 (0.52)	108	50.89 (2.52)	97	0.51 (0.12)	97	1.99 (0.29)	72
3	3.27 (0.58)	110	49.74 (2.85)	96	0.51 (0.10)	97	1.91 (0.45)	72
All	3.34 (0.56)	325	50.25 (2.92)	284	0.52 (0.11)	285	1.97 (0.36)	213

For each tertile of MGL (maternal glycemic loading), mean (sd) and *N* are reported for birth weight, birth height, weight gain per month, and height gain per month. The mean (sd) for height and weight gains reported for offspring where an assessed follow-up value existed within 4 months of the ITSEA (Infant-Toddler Social and Emotional Assessment).

## Data Availability

The datasets for this manuscript are not publicly available because human subjects were used with consent in this study, and therefore, the public release of data is not approved under the IRB. Requests to access the datasets should be directed to C.H. for approval.

## Supplementary Materials

The following are available online at https://www.mdpi.com/article/10.3390/nu13093180/s1. Figure S1: Concurrent modeling of maternal glycemic loading and Mediterranean diet on male offspring temperament. For the 3rd tertile of maternal glycemic loading compared to the 1st (referent), the odds ratio (95% confidence interval) represents the risk of being in a higher tertile of behavioral outcome. Odds ratios (95%CI) are plotted for glycemic loading (orange) and Mediterranean diet (green). Estimates were adjusted for breastfeeding at least 3 months, age of child at behavioral assessment, maternal fiber intake, total calories, folate, education, diabetes, obesity, smoking, race, and age, as well as child parity, premature birth, and weight. * *p* < 0.05, Figure S2: Concurrent modeling of maternal glycemic loading and Mediterranean diet on female offspring temperament references. Maternal glycemic loading diet and child behavior outcomes in females. For the 3rd tertile of maternal glycemic loading compared to the 1st (referent), the odds ratio (95% confidence interval) represents the risk of being in a higher tertile of behavioral outcome. Odds ratios (95%CI) are plotted for glycemic loading (orange) and Mediterranean diet (green). Estimates were adjusted for breastfeeding at least 3 months, age of child at behavioral assessment, maternal fiber intake, total calories, folate, education, diabetes, obesity, smoking, race, and age, as well as child parity, premature birth, and weight. * *p* < 0.05. Table S1: Interrogated imprint control regions and their associations with human disease and health outcomes.
