# Supplementary material for: Periconceptional Maternal Diet Characterized by High Glycemic Loading Is Associated with Offspring Behavior in NEST
Source: Nutrients. 2021 Sep 13;13(9):3180. doi: 10.3390/nu13093180 (PMC8469715; doi:10.3390/nu13093180)
Supplement: Supplementary file 1 [file nutrients-13-03180-s001.zip › nutrients-1349584-supplementary.pdf]

DMR	Location	Relevant Adverse Health Outcomes
**PLAGL1**	6q24.2	Transient neonatal diabetes mellitus, neonatal diabetes, imprinted gene network regulatory hub
**MEST**	7q32.2	Posterior cerebral artery infarction, cancer
**SGCE/PEG10**	7q21.3	Silver Russell syndrome, myoclonic dystonia (SGCE), cancer (PEG10)
**IGF2**	11p15.5	Silver Russell syndrome, Beckwith-Wiedemann syndrome, intrauterine growth restriction, obesity, metabolic syndrome, cancer
**H19**	11p15.5	Silver Russell syndrome, Beckwith-Wiedemann syndrome, cancer, imprinted gene network regulatory hub
**MEG3**	14q32.2	Age at menarche, Temple syndrome, Kagami-Ogata syndrome, recurrent pregnancy loss, autism, Parkinson's disease, osteoarthritis, cancer
**MEG3-IG**	14q32.2	Central precocious puberty, Temple syndrome, Kagami-Ogata syndrome
**NNAT**	20q11.23	Megalencephalic leukoencephalopathy with subcortical cysts 1, myoclonic epilepsy of Lafora

Table S1. Interrogated imprint control regions and their associations with human disease and health outcomes.

Concurrent Modeling of GL and MD Adjusting for All Covariates for Males

GL_T3vT1 MD_T3vT1

Domain	Outcome	GL_T3vT1 OR(95%CI)	MD_T3vT1 OR(95%CI)
Externalizing	Impulsivity	6.77 (2.08, 23.25)*	2.04 (0.66, 6.51)
24 Questions	Aggression	1.87 (0.56, 6.39)	0.81 (0.24, 2.69)
Internalizing	Depression	1.13 (0.37, 3.41)	0.90 (0.29, 2.77)
30 Questions	Anxiety	3.60 (1.05, 12.87)*	0.52 (0.16, 1.71)
	Separation	2.89 (0.92, 9.34)	1.45 (0.49, 4.33)
	Inhibition	2.78 (0.91, 8.71)	1.49 (0.49, 4.53)
Dysregulation	Emotional	1.51 (0.46, 5.04)	1.14 (0.36, 3.59)
34 Questions	Sleep	4.51 (1.43, 14.77)*	1.25 (0.40, 3.96)
	Eat	0.98 (0.31, 3.06)	0.86 (0.28, 2.60)
	Sensory	2.12 (0.63, 7.19)	1.04 (0.33, 3.26)
Compentency	Compliance	2.48 (0.78, 8.12)	0.57 (0.17, 1.82)
37 Questions	Attention	1.22 (0.40, 3.78)	2.04 (0.66, 6.38)
	Mastery	2.06 (0.64, 6.77)	1.10 (0.34, 3.53)
	Imitation	1.37 (0.42, 4.51)	0.72 (0.22, 2.29)
	Empathy	5.76 (1.66, 21.36)*	0.49 (0.14, 1.69)
	Prosocial	2.84 (0.84, 10.13)	1.67 (0.51, 5.56)
10 Questions	Social.Rel.	2.78 (0.89, 9.02)	4.48 (1.46, 14.44)*
8 Questions	Atypical	0.89 (0.27, 2.89)	0.56 (0.18, 1.71)
13 Questions	Maladaptive	10.47 (2.87, 41.36)*	0.93 (0.28, 3.08)
12 Questions	ASD Index	0.50 (0.15, 1.55)	0.49 (0.15, 1.51)

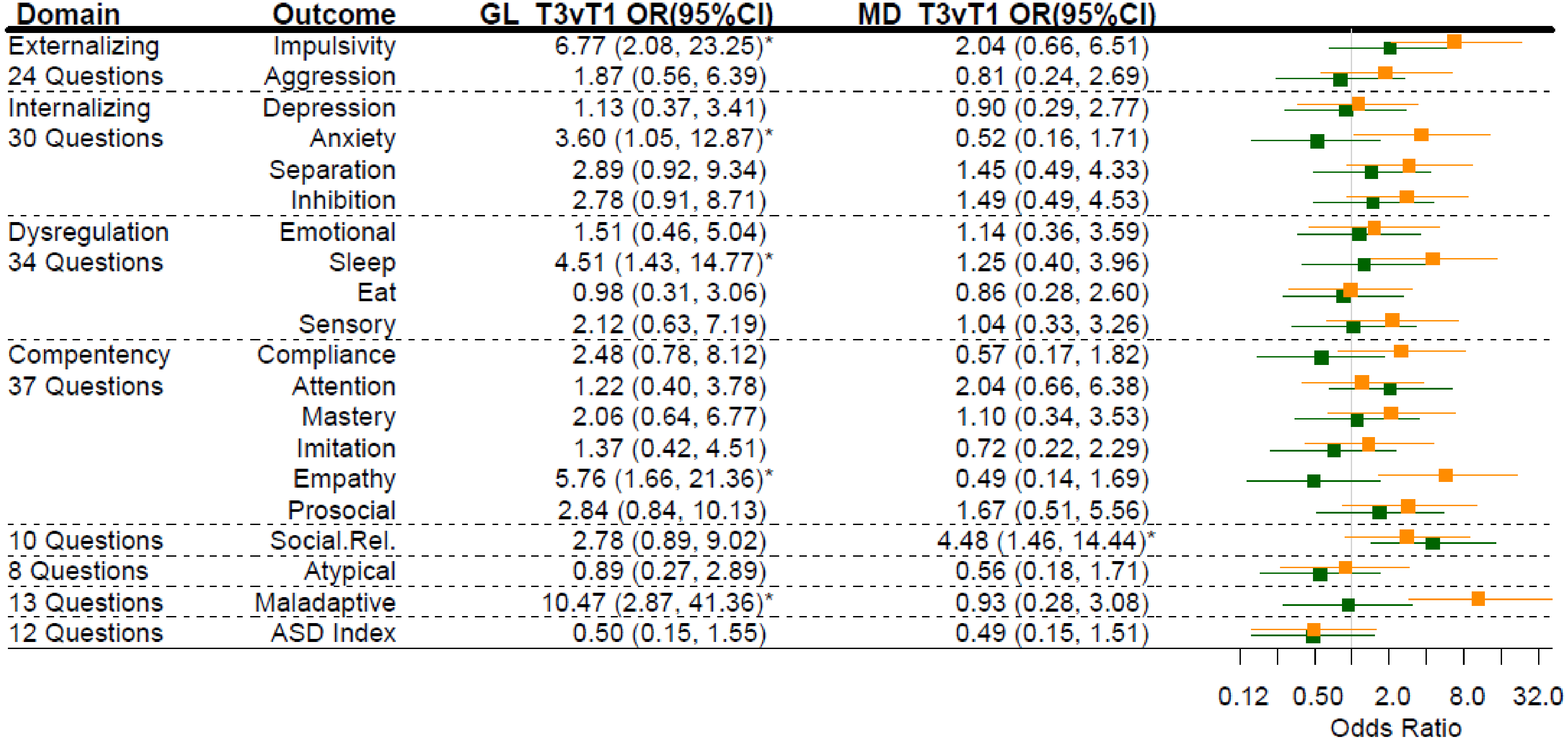

Figure S1. Maternal Glycemic Loading Diet and Child Behavior Outcomes in Males. For the 3rd tertile of maternal Glycemic Loading compared to the 1st (referent), the odds ratio (95% confidence interval) represents the risk of being in a higher tertile of behavioral outcome. Odds ratios (95%CI) are plotted for glycemic loading (orange) and Mediterranean diet (green). Estimates were adjusted for breastfeeding at least 3 months, age of child at behavioral assessment, maternal fiber intake, total calories, folate, education, diabetes, obesity, smoking, race, and age, as well as child parity, premature birth, weight. *$p < 0.05$.

Concurrent Modeling of GL and MD Adjusting for All Covariates for Females

GL_T3vT1 MD_T3vT1

Domain	Outcome	GL_T3vT1 OR(95%CI)	MD_T3vT1 OR(95%CI)
Externalizing	Impulsivity	0.43 (0.09, 1.89)	3.65 (0.96, 14.52)
24 Questions	Aggression	2.54 (0.60, 11.09)	1.73 (0.47, 6.58)
Internalizing	Depression	1.15 (0.27, 4.99)	0.07 (0.01, 0.31)*
30 Questions	Anxiety	14.14 (2.82, 82.00)*	0.46 (0.11, 1.86)
	Separation	1.53 (0.36, 6.53)	1.42 (0.40, 5.13)
	Inhibition	5.16 (1.19, 23.73)*	1.78 (0.50, 6.51)
Dysregulation	Emotional	2.58 (0.64, 10.68)	3.66 (0.99, 14.31)
34 Questions	Sleep	0.42 (0.09, 1.81)	0.57 (0.16, 2.05)
	Eat	1.00 (0.24, 4.10)	0.27 (0.07, 0.97)*
	Sensory	2.69 (0.63, 12.05)	1.41 (0.35, 5.60)
Compentency	Compliance	2.76 (0.67, 11.91)	1.33 (0.35, 5.08)
37 Questions	Attention	0.76 (0.19, 3.01)	0.69 (0.18, 2.49)
	Mastery	0.75 (0.17, 3.29)	3.82 (1.01, 14.99)
	Imitation	0.63 (0.15, 2.59)	0.84 (0.23, 3.07)
	Empathy	1.09 (0.24, 4.87)	5.22 (1.26, 23.26)*
	Prosocial	1.27 (0.26, 6.13)	1.47 (0.38, 5.73)
10 Questions	Social.Rel.	0.81 (0.19, 3.52)	1.37 (0.37, 5.20)
8 Questions	Atypical	0.54 (0.12, 2.31)	0.17 (0.04, 0.67)*
13 Questions	Maladaptive	0.85 (0.20, 3.60)	0.19 (0.05, 0.74)*
12 Questions	ASD Index	0.47 (0.09, 2.29)	0.09 (0.02, 0.41)*

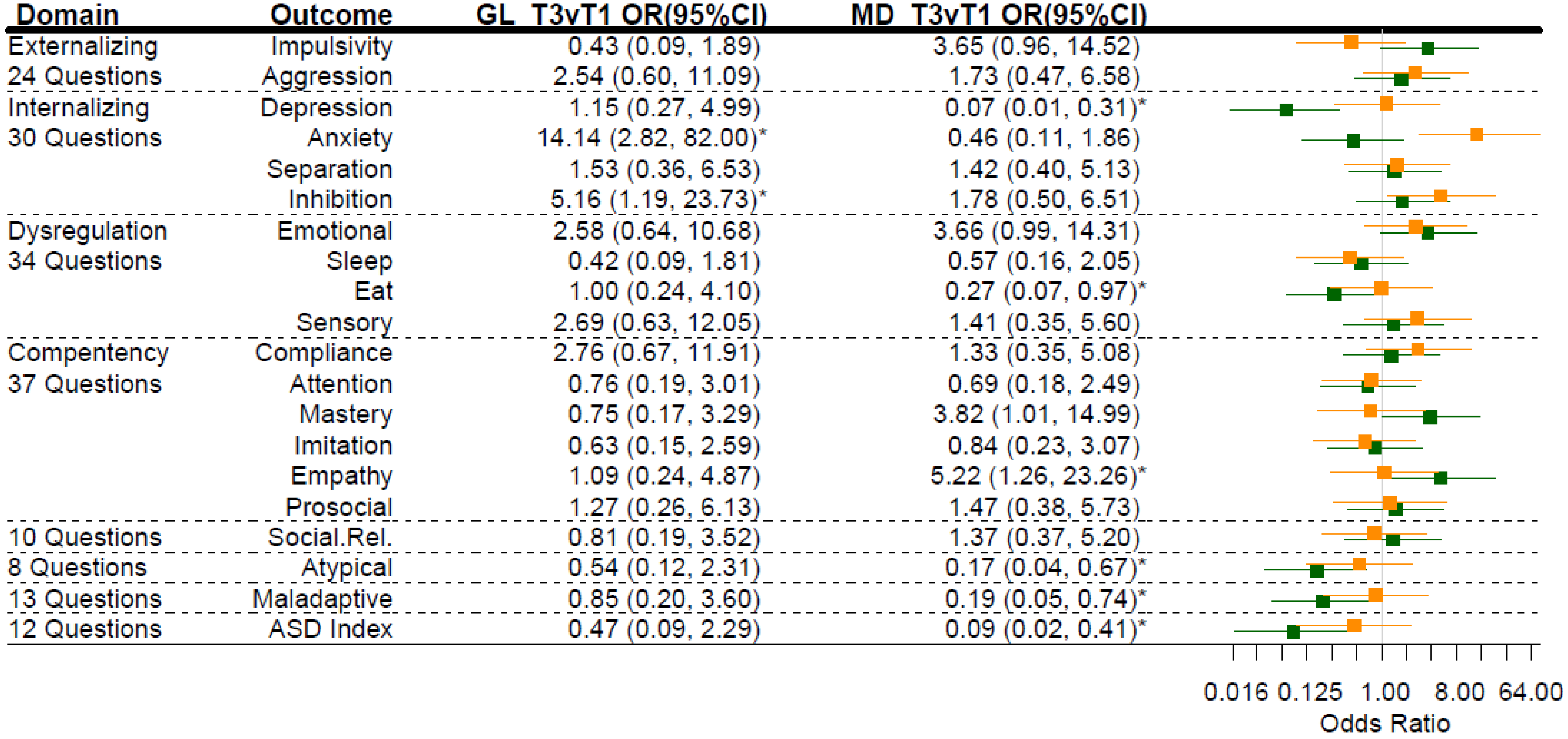

Figure S2. Maternal Glycemic Loading Diet and Child Behavior Outcomes in Females. For the 3rd tertile of maternal Glycemic Loading compared to the 1st (referent), the odds ratio (95% confidence interval) represents the risk of being in a higher tertile of behavioral outcome. Odds ratios (95%CI) are plotted for glycemic loading (orange) and Mediterranean diet (green). Estimates were adjusted for breastfeeding at least 3 months, age of child at behavioral assessment, maternal fiber intake, total calories, folate, education, diabetes, obesity, smoking, race, and age, as well as child parity, premature birth, weight. $^*p < 0.05$.
